# Pre-cooling with ingesting a high-carbohydrate ice slurry on thermoregulatory responses and subcutaneous interstitial fluid glucose during heat exposure

**DOI:** 10.1186/s40101-022-00309-w

**Published:** 2022-10-10

**Authors:** Takashi Naito, Tatsuya Saito, Akihisa Morito, Satoshi Yamada, Masatsugu Shimomasuda, Mariko Nakamura

**Affiliations:** 1 Department of Sports Research, Japan Institute of Sports Sciences, 3-15-1 Nishigaoka Kita-ku, Tokyo, 115-0056 Japan; 2 Faculty of Law, Hokkai-Gakuen University, 4-1-40 Asahimachi Toyohira-ku, Sapporo City, Hokkaido 062-8605 Japan; 3 Faculty of Medicine, Tottori University, 4-101 Koyamachominami, Tottori City, Tottori 683-8550 Japan; 4 Health Science Research R&D Laboratories, Taisho Pharmaceutical Co., Ltd., 1-403 Yoshinomachi Kita-ku, Saitama City, Saitama 331-9530 Japan; 5 Research & Development Headquarters, Self-Medication, Taisho Pharmaceutical Co., Ltd., 3-24-1, Takada, Toshima-ku, Tokyo, 170-8633 Japan

**Keywords:** Hot environments, Rest, Core temperature, Muscle temperature, Cooling strategy

## Abstract

The purpose of this study was to compare the effects of ingesting ice slurries with two different carbohydrate contents on body temperatures and the subcutaneous interstitial fluid glucose level during heat exposure. Seven physically active men underwent one of three interventions: the ingestion of 7.5 g/kg of a control beverage (CON: 26°C), a normal-carbohydrate ice slurry (NCIS: −1°C), or a high-carbohydrate ice slurry (HCIS: −5°C). The participants were monitored for a 120-min period that included 10 min of rest, 25 min of exposure to the experimental cooling intervention (during which the beverage was ingested), and 85 min of seated rest in a climate chamber (36°C, 50% relative humidity). The rectal temperature in the HCIS and NCIS trials was lower than that in the CON trial from 40 to 75 min. The infrared tympanic temperature was also lower in the HCIS and NCIS trials than in the CON trial from 20 to 50 min, whereas the deep thigh or mean skin temperatures were not significantly different among the three groups. From 90 to 120 min, the subcutaneous interstitial fluid glucose level in the NCIS trial was lower than that at 65 min; however, reductions were not seen in the HCIS and CON trials. These findings suggest that both HCIS ingestion and conventional NCIS ingestion were effective cooling strategies for reducing thermal strain, while HCIS ingestion may also enable a higher subcutaneous interstitial fluid glucose level to be maintained, ensuring an adequate supply of required muscle substrates.

## Introduction

Recently, the ingestion of an ice slurry before exercise or between exercise intervals has attracted attention as a practical cooling method, mainly in the field of sports [1–3]. The ingestion of a mixture of ice and water not only delays elevations in core temperature caused by heat exposure, but also supplies water for the body to prevent dehydration as a result of sweating during exercise. Indeed, the ingestion of an ice slurry has been demonstrated to lower the core temperature by 0.3–0.5°C [4, 5] and to improve endurance exercise performance or intermittent exercise performance, such as that required for soccer or tennis, compared with the ingestion of cold or warm water [1, 2].

Previous studies examining ice slurry ingestion as a pre-cooling strategy have focused on the timing of ingestion [6], the volume [7], or the basic mechanism (i.e., brain temperature [8] or net heat loss [9]), but the effects of the contents of ice slurries have not been studied. Clarifying how the contents of ice slurries can be optimized for physiological responses or exercise performance is of critical importance. In a previous study, an ice slurry with a temperature of −1°C was made using a commercially available sports drink containing electrolytes in addition to approximately 4–8% carbohydrates [10, 11]. The use of a high-carbohydrate ice slurry (HCIS) containing electrolytes may enable an internal cooling technique.

The following two mechanisms can explain how HCIS ingestion is effective for exercise during hot conditions. One potential rationale is that a higher carbohydrate content (higher molecular concentration) reduces the freezing point, allowing an ice slurry with a lower temperature to be created. The ice slurry would then allow more heat to be transferred from the body into the drink as a result of the enthalpy of fusion [12]. In addition to this phenomenon, because HCIS has a lower temperature than a conventional ice slurry, HCIS might be effective for attenuating increases in core temperatures.

Another potential rationale is that a high carbohydrate content would ensure an adequate supply of muscle substrates required to meet the demands of high exercise intensities and volumes [13]. The higher the exercise intensity, the higher the blood glucose utilization capacity; furthermore, a high carbohydrate intake can inhibit the blood glucose depression that causes fatigue [14]. The recommended carbohydrate intake during exercise is 30–60 g/h in terms of the blood glucose consumption rate [13], and a high carbohydrate intake within this range is likely to be useful. However, a high carbohydrate intake often causes a significant increase or decrease in blood glucose levels (i.e., blood glucose spikes) [15]. Therefore, detailed and prolonged monitoring of the effect of HCIS on blood glucose levels is needed. We focused on the subcutaneous interstitial fluid glucose (SIFG) level using a continuous glucose monitoring system as an index for the blood glucose level. This system does not require repeated blood sampling and allows for the detailed, long-term, and simple detection of peaks and drops in the blood glucose level [16]. Although a time lag between SIFG fluctuations and the actual blood glucose level does exist, SIFG is generally thought to be an acceptable indicator for blood glucose control [17].

The purpose of the present study was to compare the effects of ingesting a low- or high-carbohydrate ice slurry as pre-cooling on reducing thermal strain and to examine the effects on subcutaneous interstitial fluid glucose levels during heat exposure. We hypothesized that the ingestion of a high-carbohydrate ice slurry would reduce the core temperature and allow the maintenance of higher subcutaneous interstitial fluid glucose levels. This study was investigated at rest excluding exercise performance in order to examine in detail the core temperature and SIFG in the different volumes of carbohydrate ice slurry as pre-cooling.

## Methods

### Participants

Seven non-heat-acclimatized physically active men (age, 30.4 ± 2.7 years; height, 1.739 ± 0.049 m; body mass (BM), 70.95 ± 5.90 kg) were recruited for this study. The participants had completed a minimum of 6 h of training per week at the time of study enrollment. All the participants were non-smokers and normotensive, had no known autonomic dysfunction or cardiovascular disease, and were not taking any medications.

### Experimental protocol

Throughout the study period, the participants were asked to maintain their normal lifestyle activities at a stable level, including their physical activity and nutritional habits. During the 24-h period before the experimental trial, the participants were instructed to avoid strenuous exercise as well as the consumption of alcohol, caffeine, nonsteroidal anti-inflammatory drugs, and nutritional supplements. Each participant arrived at the laboratory after having refrained from eating for 6 h and from drinking any type of beverage for 2 h. For each participant, the three trials were commenced at the same time to control for circadian variations in the body temperature. Each trial was conducted at least 5 days apart.

**Fig. 1:**
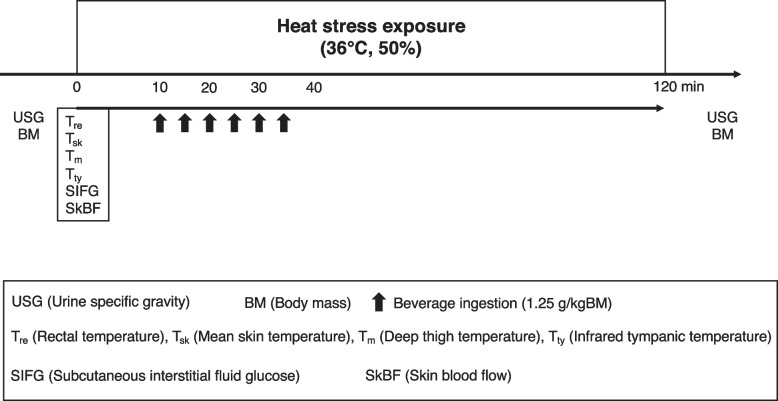
Schematic representation of the experimental protocol

Upon arrival at the laboratory, the urine samples were collected and weighed. After the measurement instrumentation was affixed in a thermoneutral condition (20–25°C, 50% relative humidity (RH)), the participants then entered the climate chamber (36°C, 50% RH). The participants remained within the climate chamber for a 120-min period that included 10 min of rest, 25 min of exposure to the experimental cooling strategy (beverage ingestion), and 85 min of seated rest. After the 85-min seated rest, the participants dried themselves with a towel and were weighed again to determine their BM before the collection of a urine sample (Fig. 1).

### *Cooling strategy*

The participants performed one of three interventions: ingestion of 7.5 g/kg BM of a control beverage (26°C, CON), a normal-carbohydrate ice slurry (−1°C, NCIS), or a high-carbohydrate ice slurry (−5°C, HCIS). To standardize the ingestion rates, the participants were given 6 boluses of 1.25 g/kg BM of the test beverage every 5 min [7]. The CON and HCIS beverages contained 79 kcal and 15.1 g of carbohydrates per 100 g, while the NCIS beverage contained 19 kcal and 4.7 g of carbohydrates per 100 g. The sodium chloride equivalent of all three beverages was 0.1 g/100 g. The same beverages as HCIS were also used in the CON trial to examine differences from the ice slurry with high carbohydrates. Both ice slurries were made using a slurry machine (Big Biz1; FMI, Japan).

### Measurements

Urine samples were used to evaluate the hydration status by measuring the urine-specific gravity (USG) before and after exercise, which was determined using a digital USG scale (PAL-09S; Atago, Japan). The nude BM was measured to the nearest 10 g using a body mass scale (HW-100KGV; A and D, Japan) before and after entering the chamber. Throughout the three trials, the participants self-inserted a rectal probe (ITP010-11; Nikkiso-Therm Co., Ltd., Japan) approximately 150 mm past the anal sphincter. The rectal temperature (Tre) was continuously recorded via a data logger (N542R; Nikkiso-Therm Co., Ltd., Tokyo, Japan) and logged intermittently at 1-min intervals. Three temperatures (chest, forearm, and thigh) were also recorded via iButtons® (Thermocron SL type; KN Laboratory, Japan) that were affixed using hypoallergenic polyacrylate adhesive tape. The mean skin temperature (Tsk) was calculated using the following formula from Roberts et al. (1997) [18]: Tsk = 0.43 × (chest temperature) + 0.25 × (forearm temperature) + 0.32 × (thigh temperature). Tty was intermittently measured three times at 5-min intervals using an infrared tympanic thermometer (ThermoScan 5 IRT4520 Ear Thermometer; Braun, Kronberg, Germany), and the average value was used.

The deep thigh temperature (Tm) was measured using a deep body temperature monitor (CM-210; Terumo Co., Ltd., Japan), which detects the tissue temperature at 5–10 mm below the skin surface using the zero-heat flow method [19]. This monitor measures the skin surface temperature beneath a thermal insulating pad containing a heater, which equilibrates the skin temperature with the deep tissue temperature when the heat flow from the skin is maintained at zero. The consistency between muscle temperature measured using a needle thermocouple and the zero-heat flow method has been evaluated previously [20, 21].

In this study, the blood glucose values were estimated using SIFG. Although a time lag between SIFG fluctuations and the blood glucose level exists, SIFG is generally thought to be an acceptable indicator for blood glucose control [17]. A calibrated disposable SIFG sensor (FreeStyle Libre sensor; Abbott Diabetes Care, Alameda, CA) was inserted and worn on the back of the upper arm for at least 1 h before the measurement. SIFG was recorded using the FreeStyle Libre Flash Glucose Monitoring System (Abbott Diabetes Care, Alameda, CA) at 5-min intervals.

Skin blood flow (SkBF) was measured using laser Doppler flowmetry using the Periflux System 5000 (Perimed, Stockholm, Sweden) and its associated software Perisoft (Perimed), which enables continuous SkBF recording (mV). Laser Doppler flowmetry provides continuous real-time measurement of local microcirculatory blood flow. A probe was attached to the frontal plane with adhesive tape. SkBF waveforms were sampled at 1000 samples per second by connecting each device to a computer using an A/D converter (PowerLab 8/35; AD Instruments, Dunedin, New Zealand). SkBF was averaged from 1 min before the measurement point.

Ratings of subjective thermal sensation (TS) were recorded using a 9-point scale ranging from 1 (very cold) to 9 (very hot) [22], while ratings of subjective comfort (RTC) were recorded using a 7-point scale ranging from 1 (very uncomfortable) to 7 (very comfortable) [23]. Recordings were made at 0, 20, 30, 40, 70, and 100 min.

### Statistical analysis

Descriptive data are presented as means ± standard deviations. All statistical computations were performed using the IBM SPSS Statistics 28 software package (SPSS, Inc., Chicago, IL, USA). The distribution of the data was analyzed using a Shapiro-Wilk test, as well as Mauchly’s test to examine the sphericity. In all cases, a two-way (drink × time) repeated-measures analysis of variance (ANOVA) was performed to compare the data for the different experimental conditions. When a significant main effect or interaction effect was identified, the differences were delineated using a Bonferroni adjustment. Pearson’s correlation coefficients were calculated to assess possible correlations between thermoregulatory responses. The strength of the correlation was defined as follows: very weak, |0.00–0.19|; weak, |0.20–0.39|; moderate, |0.40–0.59|; strong, |0.60–0.79|; and very strong, |0.80–1.0| [24]. The significance level was set a priori at *P* <0.05.

## Results

The ingestion volume was 533 ± 38 g in the CON trial, 536 ± 41 g in the NCIS trial, and 534 ± 42 g in the HCIS trial. Neither an interaction (BM: USG) nor a main effect of the trial was apparent for BM or USG (Table 1).

**Table 1:** The hydration state before and after experiment. Values are expressed as means ± SD (*n* =7)

	CON		NCIS		HCIS	
	Before	After	Before	After	Before	After
**Body mass (kg)**	71.18 ± 5.40	71.24 ± 5.41	71.10 ± 5.86	71.29 ± 5.88	71.35 ± 5.99	71.78 ± 6.41
**Urine-specific gravity**	1.019 ± 0.007	1.026 ± 0.005	1.019 ± 0.005	1.014 ± 0.006	1.019 ± 0.008	1.023 ± 0.008

### Thermoregulatory responses

**Fig. 2:**
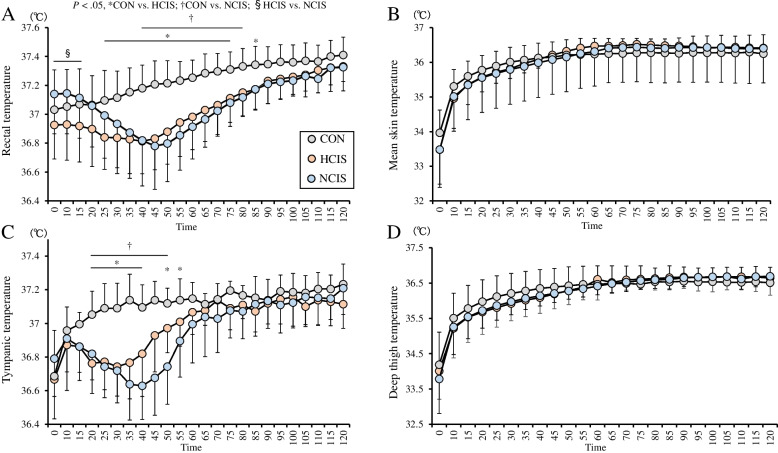
The rectal temperature (**A**), mean skin temperature (**B**), tympanic temperature (**C**), and deep thigh temperature (**D**) under three experimental conditions. The mean values are expressed as mean ± SD. Time × drink effect CON vs. HCIS: *P < 0.05, CON vs. NCIS: ^†^*P* < 0.05, HCIS vs. NCIS: ^§^*P* < 0.05

A two-way interaction in Tre was seen in the trials (*P* = .001, *F*[46,276] = 9.499). From 0 to 15 min, the Tre was higher in the NCIS trial than in the HCIS trial (*P* < .05), whereas the difference disappeared at 20 min. The Tre was lower in the HCIS trial than in the CON trial from 25 to 75 min and at 85 min (*P* < .05). From 40 to 80 min, the Tre was lower in the NCIS trial than in the CON trial (*P* < .05; Fig. 2A). In each trial, there were no significantly differences in Tre between 0 min (baseline) and each time point.

A two-way interaction (*P* = .005, F[46,276] = 1.697) in Tsk was seen in the trials, but no significant differences among the trials were seen (Fig. 2B). A two-way interaction in Tty was seen in the trials (*P* = .001, *F*[46,276] = 7.147). The Tty was lower in the HCIS trial than in the CON trial from 20 to 40 min and at 50 and 55 min (*P* < .05). The Tty was lower in the NCIS trial than in the CON trial from 20 to 50 min (*P* < .05; Fig. 2C). A two-way interaction (*P* = .001, *F*[46,276] = 3.849) in Tm was seen in the trials, but no significant differences among the trials were seen (Fig. 2D). Tre was correlated with Tty (*r* = 0.612, *P* = 0.001) and Tm (*r* = 0.492, *P* = 0.001).

### Subcutaneous interstitial fluid glucose

**Fig. 3:**
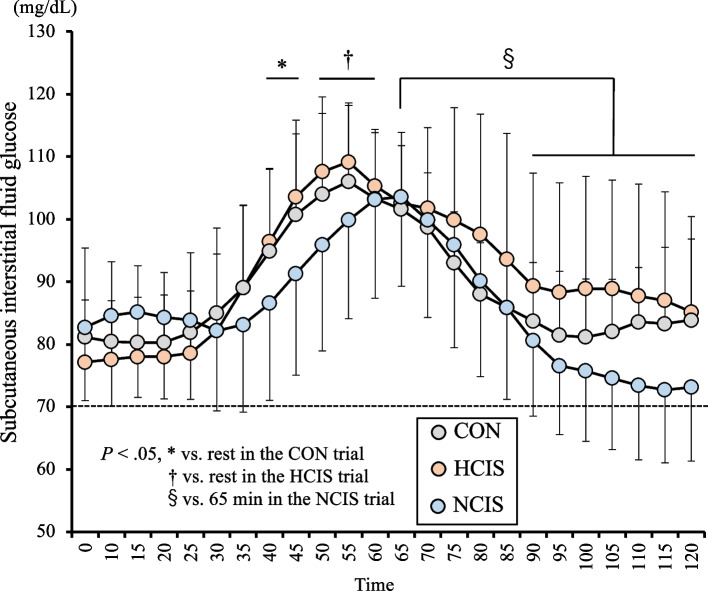
The subcutaneous interstitial fluid glucose under three experimental conditions. The mean values are expressed as mean ± SD. Dotted line reported by The American Diabetes Association [25]. Time × drink effect vs. in the CON trial: *P < 0.05 vs. in the HCIS trial: ^†^*P* < 0.05 vs. 65 min in the NCIS trial: ^§^*P* < 0.05

A two-way interaction (*P* = .001, *F*[46,276] = 2.184) in SIFG was seen in the trials. The SIFG after individual beverage ingestion increased relative to the value at rest (*P* < .05) in the HCIS (at 50–60 min) and CON (at 40–45 min) trials; however, no increase was seen in the NCIS trial. In the NCIS trial, the SIFG was lower from 90 to 120 min than at 65 min; however, no reduction was seen in the HCIS and CON trials (Fig. 3).

### Skin blood flow velocity

A two-way interaction (*P* = .020, *F*[46,276] = 1.53) in SkBF was seen in the trials, but no significant differences among the trials were seen.

### Perceptual responses

A two-way interaction in TS (*P* = .001, *F*[10,60] = 4.292) and TC (*P* = .001, *F*[10,60] = 5.772) was seen in the trials. The TS was lower in the HCIS trial from 30 to 40 min (*P* < .05) and in the NCIS trial from 20 to 70 min (*P* < .05) than in the CON trial. The TC was higher in the HCIS and NCIS trial from 20 to 40 min (*P* < .05) than in the CON trial (Table 2).

**Table 2:** Ratings of thermal sensation and comfort during experiment. Values are expressed as means ± SD (*n* =7)

	Time	0 min	20 min	30 min	40 min	70 min	100 min
**TS**	**CON**	7.0 ± 0.9	7.6 ± 0.7	8.0 ± 0.5	8.1 ± 0.4	8.3 ± 0.5	8.3 ± 0.5
	**NCIS**	7.1 ± 1.1	6.5 ± 0.8†	6.1 ± 1.1†	6.3 ± 0.9†	7.6 ± 0.5†	8.4 ± 0.5
	**HCIS**	6.9 ± 1.1	6.3 ± 1.5	6.4 ± 1.4*	6.8 ± 1.0*	7.9 ± 1.0	8.3 ± 0.7
**TC**	**CON**	3.1 ± 0.6	2.5 ± 0.8	2.0 ± 0.8	2.1 ± 0.6	2.0 ± 0.5	1.9 ± 0.6
	**NCIS**	2.9 ± 0.6	3.4 ± 0.5†	3.5 ± 0.5†	3.5 ± 0.5†	2.4 ± 0.7	1.8 ± 0.7
	**HCIS**	3.4 ± 0.7	3.8 ± 1.0*	3.3 ± 1.0*	3.1 ± 1.0*	2.3 ± 0.7	2.1 ± 0.8

Time × drink effect CON vs. HCIS: *P < 0.05, CON vs. NCIS: ^†^*P* < 0.05

## Discussion

This study compared the effects of the ingestion of a high- or normal-carbohydrate ice slurry on various body temperatures and SIFG during heat exposure. In accordance with our hypothesis, the SIFG in the HCIS trial was higher after beverage ingestion than at 0 min. On the other hand, the SIFG in the NCIS trial did not increase after beverage ingestion and was lower at 90 to 120 min than at 65 min. Unexpectedly, the Tre and Tty values, which are indicators of core temperature, were not lower during the HCIS trial, compared with during the NCIS trial.

This study is the first to compare ice slurries at different temperatures (HCIS, −5°C vs. NCIS, −1°C). There are two mechanisms by which ice slurries decrease core temperature: enthalpy of fusion [26] and conductive cooling caused by the low temperature via thermodynamic characteristics [8]. A higher carbohydrate content (higher molar concentration) allows the creation of ice slurries with a lower temperature because the freezing point is lowered. In the present study, we used a low-temperature ice slurry with a high carbohydrate content and attempted to decrease the core temperature, compared with the use of a conventional ice slurry, by conductive cooling. When 7.5 g/kg of ice slurry was ingested at sitting rest, the change in Tre in the HCIS trial was not cooler than that in the NCIS trial. Compared with the control beverage (26°C) trial, however, the absolute Tre and Tty values were both lower in the HCIS and NCIS trials, regardless of the carbohydrate contents of the beverages. As discussed by Siegel et al. [12], although the difference in drink temperatures was approximately 4°C, the large difference in Tre or Tty observed between conditions was likely due to the enthalpy of fusion rather than to the difference in drink temperature per se due to low temperature via thermodynamic characteristics. In addition, the difference between the HCIS and NCIS trials could be attributed to the change in the balance between a lower temperature (solute content) and the water content arising from the freezing point depression, worsening the efficiency of enthalpy of fusion. Since the water content and the solute contents in this study differed between the NCIS and HCIS trials, future studies should examine the effects of ice slurry ingestion at a lower temperature (solute content) on core body temperature by matching the water content between the groups.

The ingestion of a low-temperature ice slurry (HCIS, −5°C) resulted in the same reductions in the absolute of Tre and Tty as the ingestion of a conventional ice slurry (NCIS, −1°C). However, the timing of the reductions differed; the Tty decreased first, followed by a reduction in Tre. The rectum and tympanum are commonly used to measure core temperature during laboratory or field-based exercise studies. In previous studies, the Tre index was slow to respond, exhibiting a lag when compared with other measures of core temperature during thermal transitions [27]. To date, Tre and Tty have not been measured simultaneously in studies of ice slurry ingestion, but the temperature at the forehead, which is closer to the tympanum, also decreased at an earlier time point, compared with the rectal temperature [28]. The ingestion of ice slurries via the mouth may result in early conductive cooling of the facial skin, tympanum and brain [29], in turn improvement of perceptual sensation, but it did not affect the improvement of SkBF in the forehead area, which can have a positive influence on cognitive function. On the other hand, a strong correlation between Tre and Tty was observed in this study, which did not involve exercise loading, but the Tty value can be expected to vary depending on the measurement conditions. A previous study considered that a difference of ± 0.27°C between the Tre and other body sites was acceptable [30]. This temperature difference is not exceeded under moderate solar radiation [31], but it is difficult to use Tty during intense exercise in hot conditions or when the hyperthermia is high enough to cause exertional heat stroke [32]. In the present study, however, the Tty was useful for monitoring body cooling, albeit with a lag.

HCIS ingestion was predicted to have the potential to decrease core and various body temperatures. In this context, a rapid decline in Tm can impair instantaneous exercise performance. Mohr et al. [33] reported that a reduction in Tm during half-time in soccer impaired the performance of high-intensity exercise immediately after the start of the second half. Although no decrease in Tsk was observed, as in previous studies of ice slurry ingestion [28], the Tm at deeper layers also did not decline, regardless of the temperature of the ice slurries used in this study. Therefore, internal cooling, such as ice slurry ingestion, appears to avoid a reduction in active muscle temperature. On the other hand, a moderate decrease in Tm can be beneficial for exercise performance. Castle et al. [34] demonstrated that thigh cooling with an ice pack reduced Tm measured with a needle probe by approximately 3.5°C from 36.5 to 33°C, in turn improving subsequent intermittent exercise performance. In addition, Hasegawa et al. [35] recently reported that a 12°C-ice pack decreased the Tm by approximately 3°C and attenuated the impairment to subsequent power output during intermittent sprint performance, albeit no improvement was observed using a 0°C-ice pack. Thus, combined cooling using both ice slurry ingestion and moderate cooling of the muscles may be an effective strategy during heat exposure.

In the HCIS trial, the SIFG increased after beverage ingestion, but no difference was seen compared with the NCIS trial. The volumes of carbohydrate intake were approximately 80 g in the HCIS trial and 25 g in the NCIS trial. These results were consistent with the results of studies by Short et al. [36] and Jentjens et al. [37], which did not observe a difference in blood glucose concentrations at 45–60 min after the intake of between 75 and 25 g of carbohydrates. In the second half of the trial, the NCIS trial dropped to near 70 mg/dL, which have defined as low blood glucose (hypoglycemia) by The American Diabetes Association [25]. This threshold has also been used for SIFG in a previous study [38]. Since this reduction was not observed in the HCIS trial, the amount of carbohydrates, rather than the cooling intervention achieved through the ingestion of an ice slurry during heat exposure, was likely involved. Serum insulin has a significant effect on blood glucose or SIFG control at rest, and carbohydrate intake after fasting increases serum insulin secretion [39]. The serum insulin concentration might have affected the reduction in SIFG in the NCIS trial as well as in previous studies [36, 37]. However, serum insulin has somewhat individual differences in sensitivity and secretory capacity. For example, a higher aerobic capacity is associated with greater insulin sensitivity [40]. In this study, higher SIFG values were maintained during the HCIS trial without a significant decrease, although factors associated with insulin were not examined.

### Limitations

There are some limitations associated with the present study. First, this study used the SIFG, not the blood glucose level, and did not measure the serum insulin concentration. Direct measurements of blood glucose and the serum insulin concentration require blood sampling via needle puncture, which is invasive and can cause the subject distress. To measure the glucose concentrations in a continuous and noninvasive manner, this study focused on the SIFG using a continuous glucose monitoring system as an index for the blood glucose level. Although this system has a time lag between SIFG fluctuations and the actual blood glucose level, SIFG is generally thought to be an acceptable indicator for blood glucose control [17]. In the present study, the ability to continuously monitor SIFG at rest allowed us to confirm the presence or absence of blood glucose spikes associated with HCIS ingestion. Finally, the presently reported experiment was performed with the participants at rest to exclude the effects of exercise on thermoregulation and SIFG. If exercise is performed after pre-cooling with HCIS, a beverage with a high carbohydrate content would slow the rate of gastric emptying and restrict absorption from the small intestine to approximately 1.0 g/min [13]. Indeed, Hatta et al. [41] suggested that the ingestion of beverages with high carbohydrate contents before exercise did not induce hypoglycemia, compared with beverages with low carbohydrate contents, because of the slow rate of gastric emptying. During exercise, the SIFG responses may be similar to those reported in the previous study mentioned above. However, the gut is a trainable and adaptable organ, and with proper practice, athletes can improve their tolerance to exogenous carbohydrate intake [42]. Such interventions may need to be modified to suit the characteristics of individuals during practical application.

## Conclusion

This study demonstrated that the ingestion of a high-carbohydrate ice slurry decreased the rectal and infrared tympanic temperatures, compared with the ingestion of a 26°C beverage, similar to the ingestion of a low-carbohydrate ice slurry. The ingestion of a high-carbohydrate ice slurry attenuated the reduction in SIFG. These results suggest that HCIS ingestion is an effective cooling strategy for reducing thermal strain, like conventional NCIS, with the added benefit of the maintenance of a high subcutaneous interstitial fluid glucose value.

## Data Availability

Not applicable.

## Abbreviations

BM: Body mass
CON: Control beverage
HCIS: High-carbohydrate ice slurry
ISFG: Subcutaneous interstitial fluid glucose
NCIS: Normal-carbohydrate ice slurry
RH: Relative humidity
SkBF: Skin blood flow
TC: Thermal comfort
Tm: Deep thigh temperature
Tre: Rectal temperature
TS: Thermal sensation
Tsk: Mean skin temperature
Tty: Infrared tympanic temperature
USG: Urine-specific gravity
